# Microbial assembly among plant parts of a tropical tree in different habitats and seasons

**DOI:** 10.1128/spectrum.01835-25

**Published:** 2026-01-30

**Authors:** Muhammad Qaisar Naeem Khan, Wen-Qian Xiang, Ya-li Wei, Ming-Xun Ren

**Affiliations:** 1 Ministry of Education Key Laboratory for Genetics and Germplasm Innovation of Tropical Special Forest Trees and Ornamental Plants, Hainan University 74629, Haikou, China; 2 International Joint Center for Terrestrial Biodiversity around South China Sea of Hainan Province, School of Ecology, Hainan University 74629, Haikou, China; Mikrobiologický ústav AV ČR, v. v. i., Třeboň, Czech Republic

**Keywords:** plant microbiome, plant environment, ecology, *Bombax ceiba*, microbial assembly

## Abstract

Microorganisms are exhibited in every environment, but their role in the ecosystem and host association remains to be understood. Microbiome research highlights environmental filtering as a factor shaping microbial communities in terrestrial ecosystems. We analyzed bacterial and fungal communities in flowers, leaves, branches, trunks, roots, and soil of wild and rice field-grown *Bombax ceiba* under different seasons using the recent advanced techniques of 16S rRNA and ITS rRNA amplicon pyrosequencing, respectively. Our findings revealed variation in microbial communities across different plant parts and heights (flower, leaf, branch, trunk, roots, and soil). NMDS and hierarchical clustering (Bray-Curtis dis) analysis revealed that bacterial and fungal communities related to endophytic (within plant tissue) and epiphytic (external plant surfaces) niches of above and below-ground parts significantly differed due to seasonal variation in rice and wild habitats. Moreover, seasonal variation significantly affected the number of shared OTUs and bacterial and fungal taxa across all plant parts in both habitats. The spring season significantly influenced microbial diversity, assembly, and correlation networks in plant parts, with the most potent effects in the rice field compared to the wild habitat of *Bombax ceiba*, influencing flowering and boosting Firmicutes abundance. Wild habitats possessed higher functional diversity than rice-grown fields in spring and autumn across all plant parts, indicating their strong relation with different environments. The outcomes of the present study revealed that seasonal changes across various habitats dynamically shape the plant microbiome, suggesting distinct ecological roles (e.g., nutrient cycling, pathogen suppression, and stress tolerance) for bacterial and fungal communities in various niches.

**IMPORTANCE:**

This study shows that seasonal variation and habitat type (wild vs rice field) significantly influence the diversity, composition, and assembly of bacterial and fungal communities in different plant parts of *Bombax ceiba*. By analyzing both endophytic and epiphytic niches across above- and below-ground compartments, it reveals the spatial complexity of plant microbiomes. Wild habitats support greater stability to microbiome and their functions as compared with rice fields, particularly in spring, indicating that land-use practices affect microbiome structure and function. The study gives the details on how the compositions of microbiomes associated with plants change with season, which can impart insight into sustainable crop-management strategies, as well as conservation measures.

## INTRODUCTION

Although microorganisms are found in every environment, our knowledge of their function in ecosystems and about host organisms is still developing. Individual microorganisms associated with plants are known to support essential processes throughout the plant, such as the acquisition of nutrients and water, the response to stress, the suppression of diseases (1), and the direct and indirect reduction of herbivory by priming host plant defenses (2). Furthermore, studying plant-microbe relations is essential not only to study their role in the development and growth of plants but also to enable using these relationships in sustainable plant production, secondary metabolite production, and phytoremediation applications (3). To avoid the loss of woody plants, the researchers need to focus on the crucial role of plant-microbial interactions under various environmental stresses and the potential role of plant responses to these adverse environmental conditions (4). Furthermore, a deep comprehension of the ecology of plant microbial communities may have implications for bettering conservation efforts, which are becoming more and more popular (5, 6). However, knowledge of the makeup of microbiomes in distinct host environments has advanced. Still, there is limited research or knowledge on the diversity of makeup of microbiomes throughout the many possible microbial niches characterized by various plant organ and tissue types (7).

Microbial communities in terrestrial environments are primarily structured by environmental filtering, which is deterministic species-sorting processes that rely on environmental variables, according to decades of microbiome research (8). Seasonal variations in community shaping might play a significant part in the temporal variation of the plant microbiome in environmental dynamics. Additional environmental factors, including soil, climate, topography, farming practices, and plant domestication, may also contribute to variations in the makeup of plant-associated microbial communities (9). A shift in one aspect of the environment causes changes in the phenotype of plants, which in turn alters the collection of different microbiomes that contain plant compartments (10, 11). Most research on plant microbiomes has concentrated on the microbiota of agricultural crops (12, 13). However, little is understood about the microbial community assembly associated with woody plants in agricultural lands and how their microbiomes differ from those in wild habitats. Additionally, the microbiota assembly in these various environments is impacted by seasonal change. Such knowledge gaps limit our understanding of environmental patterns of microbial structure and their diversity and how they react to variations in their surroundings.

*Bombax ceiba* is exhibited worldwide and belongs to the Malvaceae family. *Bombax ceiba* characteristics include tall heights, buttress roots, and glorious red flower bearing in spring. It has great importance in landscaping architecture. In tropical regions such as Vietnam, Thailand, Bangladesh, Nepal, and south China, it is extensively dispersed among nearby rice farms and forms a vast agroforestry system (14). In China, Hainan Island has kept many ancient *B. ceiba* trees in rice fields, and rice seedlings are transplanted only once *B. ceiba* begins to blossom, typically around mid-February (15). Furthermore, its seedlings have been widely grown for financial gain due to its widespread distribution in the tropics and high demand as a plant species for sculpture (14). However, several studies discovered variations in the organic matter inputs from trees changed the diversity and composition of soil microbes in agroforestry systems (16). On the other hand, no research has been done yet to ascertain the impact of these agricultural and wild environments on the assembly of *Bombax ceiba* microbiome.

This study seeks to understand the mechanism governing how microbial communities assemble in distinct compartments of *B. ceiba*. We examined fungal and bacterial communities in above- and below-ground plant parts (flower, leaves, branches, trunks, roots) and soil across endophytic and epiphytic niches in two habitats (wild and rice field) and seasons (flowering and non-flowering). Despite growing recognition of plant-associated microbiomes, it remains unclear how vertical plant structure and seasonal dynamics jointly shape microbial assembly across endophytic and epiphytic niches. To address this gap, we hypothesized that microbial communities are organized in a vertical gradient from root to leaf, with the soil microbiome serving as the primary microbial reservoir; distinct plant parts and habitats (endophytic vs epiphytic) drive significant differences in community composition and ecological functions; and seasonal changes further restructure soil and phyllosphere microbiomes, thereby reshaping plant-associated microbial communities across niches.

## MATERIALS AND METHODS

### Study area and sampling

**Fig 1:**
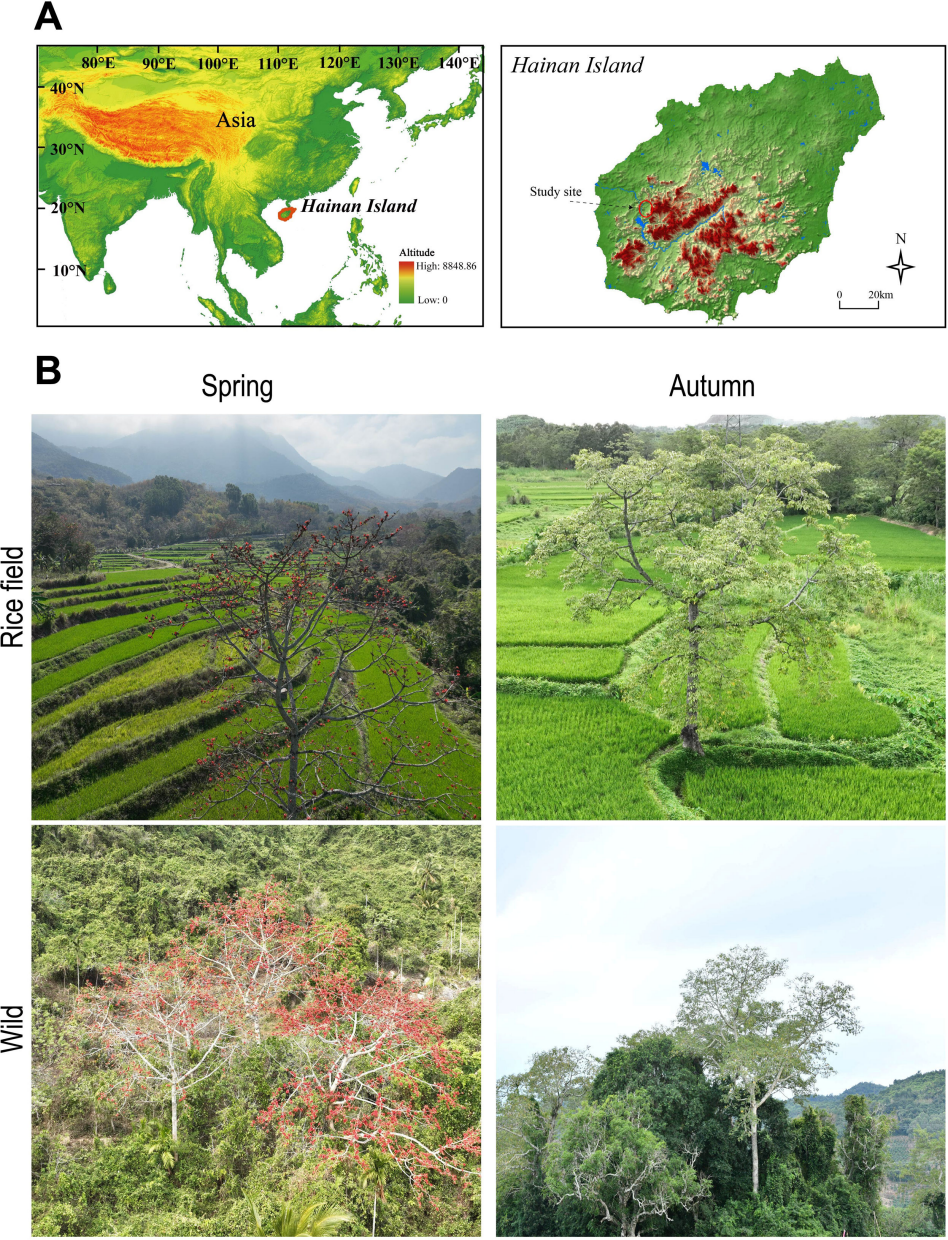
Geographical location and aerial view of *Bombax ceiba* plant grown in rice fields and wild on Hainan Island, China. (**A**) Geographic location of the study site. (**B**) Representative aerial imagery of the study area showing rice fields and wild habitat across seasons (spring and autumn).

This study site was located at Changjiang County (19.0801441°N, 109.08252198°E), Hainan Island, China (Fig. 1). We have selected two sites (wild and rice fields) for two consecutive seasons, autumn and spring. Mature trees of *B. ceiba* within >5 m distance from each other were selected. Five leaves were extracted from each of the three branches and removed from the lowest, middle, and top portions of the crown for every tree. Flower’s nectar was collected in the spring season by using a sterilized pipette and immediately placed in an ice bucket. Using a sterilized knife, the bark was cut off the tree’s trunk and branch before samples were taken. Next, 0.8- × 0.3-cm samples were extracted from the soft layer of living cells according to a previous study (17). The distinct samples were extracted from the core dead wood underneath using a 5-mm diameter sterile drill. Three heights (20, 150, and 170 cm) were used to collect trunk pairs of living and dead wood samples from a single tree. For every tree compartment, these samples were merged to produce a single composite sample. And from each part of the tree, three biological replicates were collected in each season. Root samples were collected in two parts: near the stem (coarse root) and the root tip samples. Three locations, each less than a meter from the tree stem, yielded three soil samples combined into one sample per tree in the labpratory. The nectar, wood, and soil samples were kept at −80°C for further analysis.

### Sample preparation

We have processed the leaves and roots samples in the following ways. In the laboratory, weigh the leaves and add 10 mL 0.1M sterile potassium phosphate buffer (K_2_HPO_4_ + KH_2_PO_4_, 0.1 M, pH = 8.0) to each gram of sample. Ultrasound the sample for 1 min, vortex for 10 s, and repeat this step twice. Keep the washing solution, remove the washed leaves, and repeat the above experimental steps two and three times. All washing solutions were filtered by a 0.22-μm filter membrane; the filtered filter membrane was kept in a 2.5-mL sterile tube and stored in a −80°C refrigerator. Further, the leaves were washed with sterilized water for 30 s and four times with 75% ethanol for 1 min and with 2% sodium hypochlorite solution for 3 min, after that again with 75% ethanol for 1 min and 30 s by sterile water and stored at −80°C (18).

To get rid of bulk soil that was loosely connected, the root samples were carefully removed from the soil under aseptic conditions and gently shaken. The root was vortexed thrice for 15 s in 5 mM sodium pyrophosphate to obtain the loosely bound rhizosphere soil. The root was then sonicated for 5 min to extract the firmly bound fraction in a fresh pyrophosphate buffer. The firmly and loosely bound fractions were combined for the next step of DNA extraction to provide a complete representation of the rhizosphere microbiome (19). Further, root samples were washed to extract the endophytic microorganisms, followed by the method used for leaves.

### DNA extraction and PCR amplifications

All microbes’ genomic DNA was extracted using the E.Z.N.A. soil DNA kit from Omega Bio-tek, situated at Norcross, GA, United States, following the designated protocol. A NanoDrop 2000 spectrophotometer (Thermo Fisher Scientific, United States) and 1.0% agarose gel electrophoresis were used to assess the purity and concentration of DNA, which was then stored at −80°C before further use. The bacterial 16S ribosomal DNA (rDNA) is amplified using primers 515F (GTGCCAGCMGCCGCGG) and 806R (GGACTACHVGGGTWTCTAAT). However, the ITS1F (CTTGGTCATTTAGAGGAAGTAA) and ITS2R (GCTGCGTTCTTCATCGATGC) primers for fungi were selected using the following conditions: T100 5× Fast Pfu buffer (5 μL), 2 μL 2.5 mM dNTPs, 2 μL of each ITS1 and ITS2 primer (5 μM), 3 μL Fast Pfu polymerase, 2 μL of template DNA and ddH2O up to 20. The following were the cycling conditions for PCR amplification: first denaturation for 3 min at 95°C, followed by 27 denaturing cycles for 30 s at 95°C, 30 s of annealing at 55°C and 45 s of extension at 72°C, and one 10-min extension at 72°C, and end at 4°C. The PCR product from the 2% agarose gel was then purified with the PCR Clean-Up Kit (YuHua, Shanghai, China) based on the manufacturer’s instructions, and the Qubit 4.0 (Thermo Fisher Scientific, USA) was used to measure the quantities.

### Illumina PE300/PE250

After purification, amplicons from all pools in equal concentration were subjected to the sequencing analysis on the Illumina PE300/PE250 platform (Illumina, San Diego, CA, USA) using the paired-end method with common strategies from Majorbio Bio-Pharm Technology Co. Ltd. (Shanghai, China).

### Data processing

Using an internal Perl script, raw FASTQ files were de-multiplexed. Fastp version 0.19.6 (20) then quality-filtered them, and FLASH version 1.2.7 (21) merged them based on the following standards. (i) The reads that had any location truncated at an average quality below 20 over any 50bp length window were discarded as all the reads included ambiguous characters were also excluded. (ii) Only sequences that overlapped and were longer than 10 base pairs were put together. The overlap region’s maximum mismatch ratio is 0.2. Unable-to-assemble reads were thrown away. (iii) The barcode and primers were used to identify the samples, and the sequence orientation was changed. Two nucleotide mismatches in primer matching were also taken into consideration. Subsequently, UPARSE 7.1 (22, 23) was applied to classify the optimized sequences into OTUs at a 97% sequence similarity level. For every OTU, the most prevalent sequence was chosen as a representative one. Chloroplast sequences in every sample were eliminated by manual filtering of the OTU table. Each sample’s 16S rRNA gene sequences were subset down to 20,000 sequences to minimize the effects of sequencing depth on the alpha and beta diversities. This nevertheless created an average Good’s coverage of 99.09 percent, respectively.

The taxonomy of each representative sequence of functional OTUs was checked using RDP Classifier Version 2.2 (24) with a confidence threshold level of 70% against its corresponding 16S rRNA gene database, such as Silva v138. Biologically derived OTU representative sequences were analyzed for the metagenomic function based on Phylogenetic Investigation of Communities by Reconstruction of Unobserved States (PICRUSt2). KEGG-based functional predictions prioritized microbial processes linked to nutrient cycling, energy metabolism, and ecological interactions relevant to plant health, as determined by PICRUSt2. The software program PICRUSt2 includes the following set of utilities: HMMER was then used to align the OTU representative sequences with reference sequences. OTU representative sequences were rooted into a reference tree with the help of EPA-NG and Gappa. The values were normalized using a castor to make the measurement of the 16S gene copies easier. MinPath was employed to determine these gene pathways and anticipate gene family trends. The entire analysis was performed based on OTU representative sequences and according to PICRUSt2 (25). The program PICRUSt2 includes the following set of tools: the OTU representative sequences were then compared to reference sequence databases using HMMER. PICRUSt2 protocols carried out the whole analytic procedure: MinPath was used to predict gene family trends and map to gene pathways; EPA-NG and Gappa were used to position OTU representative sequences in a reference tree; and castor was used to standardize the amplitude of 16S gene copies. Fungal taxa were classified and their ecological functions assigned using the FUNGuild database (26).

### Statistical analysis

The Majorbio Cloud platform (https://cloud.majorbio.com) was used to perform bioinformatic analysis of the plant microbiome. The alpha diversity indices, namely the observed number of OTUs, Chao1 richness, and Shannon index, were calculated from the OTUs data using Mothur v1.30.1 software as described by Schloss et al. (27). Cluster analysis was done using the NMDS technique and the Bray-Curtis dissimilarity with the vegan v 2.5-3 package. The ANOISM test was conducted to evaluate the amount of variance explained by the treatments using the vegan v2.5-3 program. To find the significantly enriched taxa of bacteria at the phylum to genera level, the linear discriminant analysis (LDA) effect size (LEfSe) analysis was done, with an LDA score of more than 2 and a *P*-value of less than 0.05. (28). These co-occurrence networks were designed to explore the internal communal connections between the samples (29). A correlation between two nodes was considered significant at a Spearman’s correlation greater than 0.6 or less than −0.6 with a probability value of less than 0.01. Three technical and biological replications have been carried out for all tested samples. The normalized stochasticity ratio (NST, based on Chao distance algorithm) was used to quantitatively evaluate whether community assembly was more deterministic (<50%) or stochastic (>50%) (30). Microbial source tracking data were also analyzed on the online tool of Majorbio Cloud Platform (https://cloud.majorbio.com/page/tools/). A partial least squares path model (PLS-PM) was established in RStudio using the plspm package (v 0.4.9) to investigate the effects of season and niche on microbial diversity (Chao and Shannon), microbial structure (NMDS1 and NMDS2), and functions (PICRUSt2 ad FUNGuild).

## RESULTS

### Core bacterial and fungal communities across different parts of *B. ceiba*

**Fig 2:**
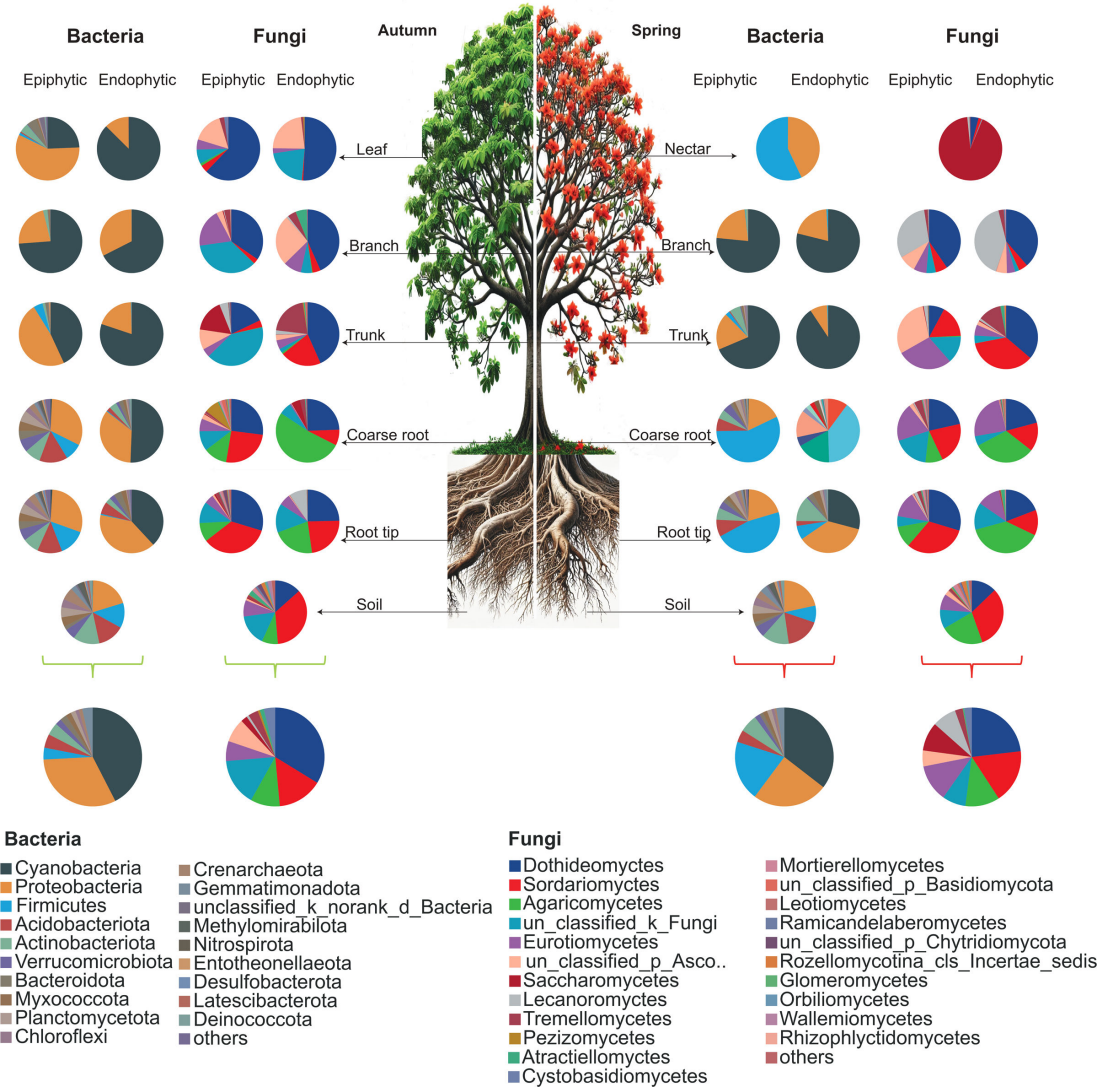
Relative abundance of the dominant bacterial phyla and fungal class averaged across endophytic and epiphytic niches of plant parts (nectar, leaf, branch, trunk, coarse root, root tip) and soil in different seasons (autumn and spring) in rice growing fields of *B. ceiba*.

**Fig 3:**
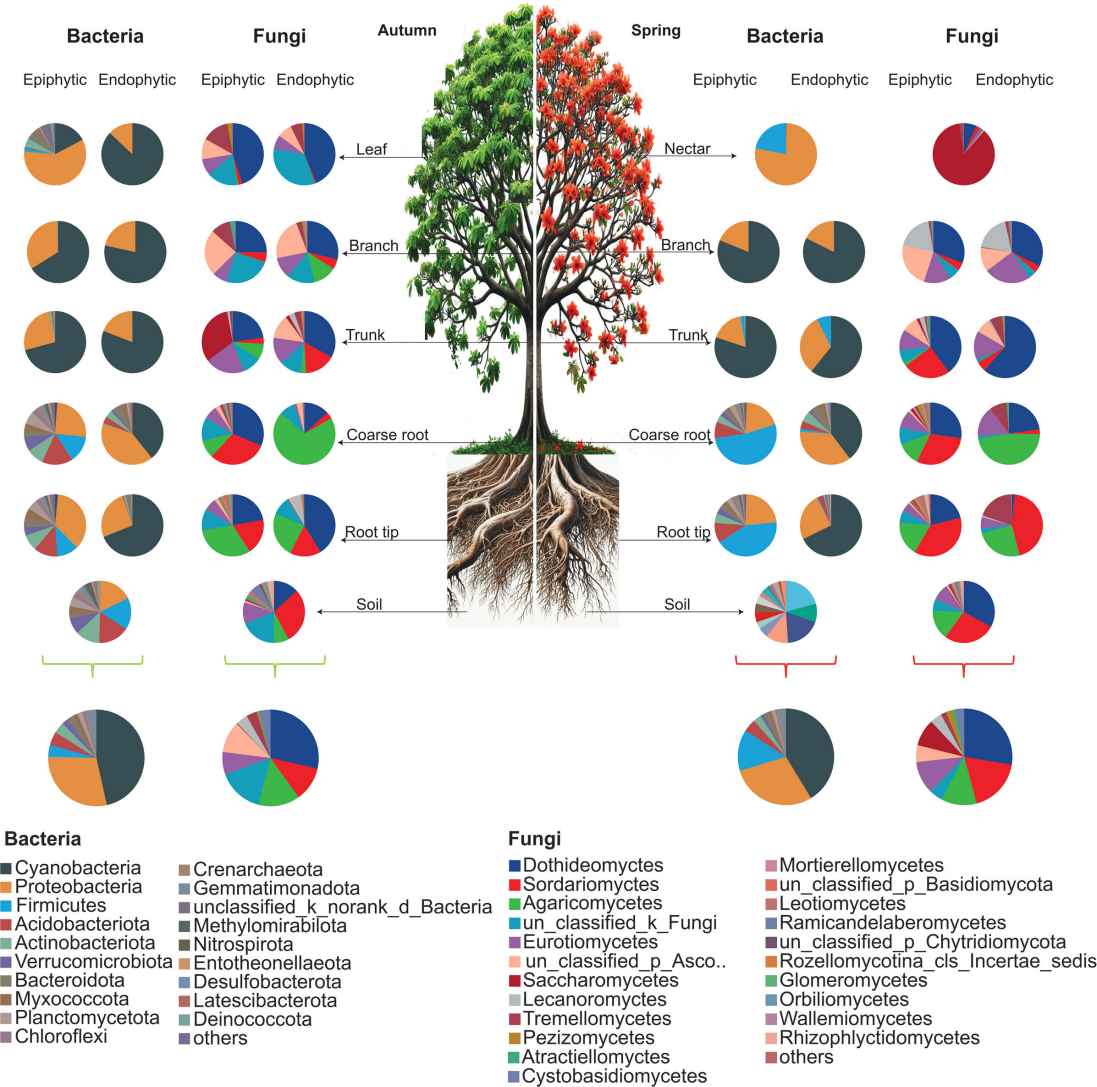
Relative abundance of the dominant bacterial phyla and fungal class averaged across endophytic and epiphytic niches of plant parts (nectar, leaf, branch, trunk, coarse root, root tip) and soil in different seasons (autumn and spring) in the wild habitat of *Bombax ceiba*.

In autumn, Cyanobacteria and Proteobacteria dominated bacterial communities in both rice (74.16%) and wild habitats (75.34%). In spring, Cyanobacteria, Proteobacteria, and Firmicutes were most abundant, comprising 79.92% (rice) and 70.3% (wild) of the total population (Fig. 2 and 3).

In both rice and wild habitats, the dominant fungal phyla were Ascomycota, Basidiomycota, and unclassified_k_fungi, comprising over 98% of the total fungal community in both autumn and spring. Ascomycota was the most abundant phylum in all conditions, with slightly higher proportions observed in spring (see Fig. S1b at https://doi.org/10.5281/zenodo.17585670).

In autumn in rice-grown *B. ceiba*, epiphytic cyanobacteria were most abundant on branches (73.8%) and trunks (43.1%), with lower presence on leaves and roots. Epiphytic Proteobacteria were highest on leaves (57.8%), trunks, and coarse roots. In endophytic niches, cyanobacteria dominated leaves (87.4%), trunks, and branches, while Proteobacteria were most abundant in root tips (40.1%), coarse roots, and branches. In soil, Proteobacteria abundance was 20.1%, and cyanobacteria were low (0.6%) (Fig. 2).

In spring, epiphytic cyanobacteria were most abundant on branches (76.5%) and trunks (68.6%), with lower presence on coarse root and root tip. Endophytic cyanobacteria peaked in trunks (90.6%) and branches (78.8%). Epiphytic Proteobacteria were highest in branches (21.6%) and root tips (20%), while soil had 0% cyanobacteria but 21.6% Proteobacteria. Firmicutes, the third most abundant phylum, were dominant epiphytically in coarse root (56.8%) and root tip (46.6%), but less abundant endophytically. Nectar had high Firmicutes (57.1%) and Proteobacteria (43%), while soil contained Proteobacteria (21.5%), Acidobacteriota (17.5%), Actinobacteria (13.9%), and Firmicutes (8.6%) (Fig. 2).

In wild habitats of *B. ceiba*, seasonal changes significantly affected overall bacterial composition across all niches. In spring, Cyanobacteria dominated (41.3%), followed by Proteobacteria (29.0%) and Firmicutes (13.8%). In autumn, Cyanobacteria abundance increased, while Proteobacteria (28.9%) and Firmicutes (4.1%) decreased compared with spring (Fig. 3).

In autumn, dominant fungal classes in rice and wild habitats were Dothideomycetes, Sordariomycetes, unclassified_k_fungi, and Agaricomycetes, comprising over 73% and 69% of the communities, respectively. In spring, Dothideomycetes, Sordariomycetes, Eurotiomycetes, Agaricomycetes, and Saccharomycetes dominated, accounting for about 74% and 77% of total fungal abundance. Overall, Dothideomycetes remained the most consistently dominant class across both seasons and habitats (see Fig. S1c at https://doi.org/10.5281/zenodo.17585670).

Fungal communities in *B. ceiba* showed significant seasonal variation across endophytic and epiphytic niches in both rice and wild habitats. In the phyllosphere, dominant fungal classes were Dothideomycetes, unclassified_k_fungi, and Sordariomycetes. In the endosphere during autumn, the main classes were Dothideomycetes, Agaricomycetes, Sordariomycetes, and unclassified Ascomycota. Soil fungal communities were richer, predominantly Sordariomycetes and unclassified_k_fungi. In spring, phyllosphere niches were dominated by Dothideomycetes and Saccharomycetes, while endosphere niches mainly had Dothideomycetes, Agaricomycetes, and unclassified_k_fungi. Soil consistently showed higher fungal diversity with predominant Sordariomycetes and Agaricomycetes. Both habitats shared similar dominant fungal classes across plant parts but differed in relative abundances (Fig. 3).

### Community assembly

The NST index revealed that bacterial communities in endophytic niches were less stochastic than in epiphytic ones. In autumn, the highest bacterial stochasticity occurred in the rhizosphere of coarse roots, root tips, and soil, while in endophytes, it was highest in root tip and coarse root, indicating greater stochasticity in below-ground parts. This pattern was similar in wild habitats, with spring soil also showing high stochasticity. Fungal communities exhibited even greater stochasticity than bacteria across all plant parts and habitats. In autumn, epiphytic niches had the highest fungal stochasticity in root tip, soil, and coarse roots; endophytes showed it in coarse root, root tip, and branch. Spring also showed high but lower stochasticity, with soil and coarse roots leading epiphytic niches, and coarse root and root tip in endophytes. Below-ground parts and epiphytic niches were generally more stochastic, and fungal communities in wild habitats were consistently more stochastic than those in rice fields (see Fig. S2 at https://doi.org/10.5281/zenodo.17585670).

### Microbial diversity and structure among different parts of *B. ceiba*

Alpha diversity analysis showed that seasonal changes strongly influenced bacterial and fungal richness and diversity across endophytic and epiphytic niches of all plant parts in both wild and rice field habitats of *Bombax ceiba*. Bacterial Chao richness and Shannon diversity were generally higher in rice fields than in wild habitats during both autumn (2,153 ± 242.7; 3.1 ± 0.3 vs 1,794.4 ± 163.7; 2.8 ± 0.23) and spring (1,773.9 ± 235.5; 2.8 ± 0.24 vs 1,527.8 ± 237.3; 2.4 ± 0.36) (see Fig. S3a and b at https://doi.org/10.5281/zenodo.17585670).

Fungal Chao richness was higher in rice-grown *B. ceiba* during both autumn (684.86 ± 66) and spring (440.8 ± 126.4) compared with wild habitats (598 ± 65.6 and 364.5 ± 62.4, respectively). However, fungal diversity was slightly lower in rice fields (3.24 ± 0.29 in autumn, 2.8 ± 0.5 in spring) than in wild habitats (3.35 ± 0.3 in autumn, 2.9 ± 0.27 in spring). Overall, autumn showed higher bacterial and fungal richness and diversity than spring in both habitats (see Fig. S3c and d at https://doi.org/10.5281/zenodo.17585670).

Furthermore, alpha diversity analysis showed that the bacterial and fungal communities significantly vary at both niches of all plant parts as compared to soil with respect to season in both rice fields and wild of *B. ceiba,* as shown in (see Fig. S4 at https://doi.org/10.5281/zenodo.17585670).

**Fig 4:**
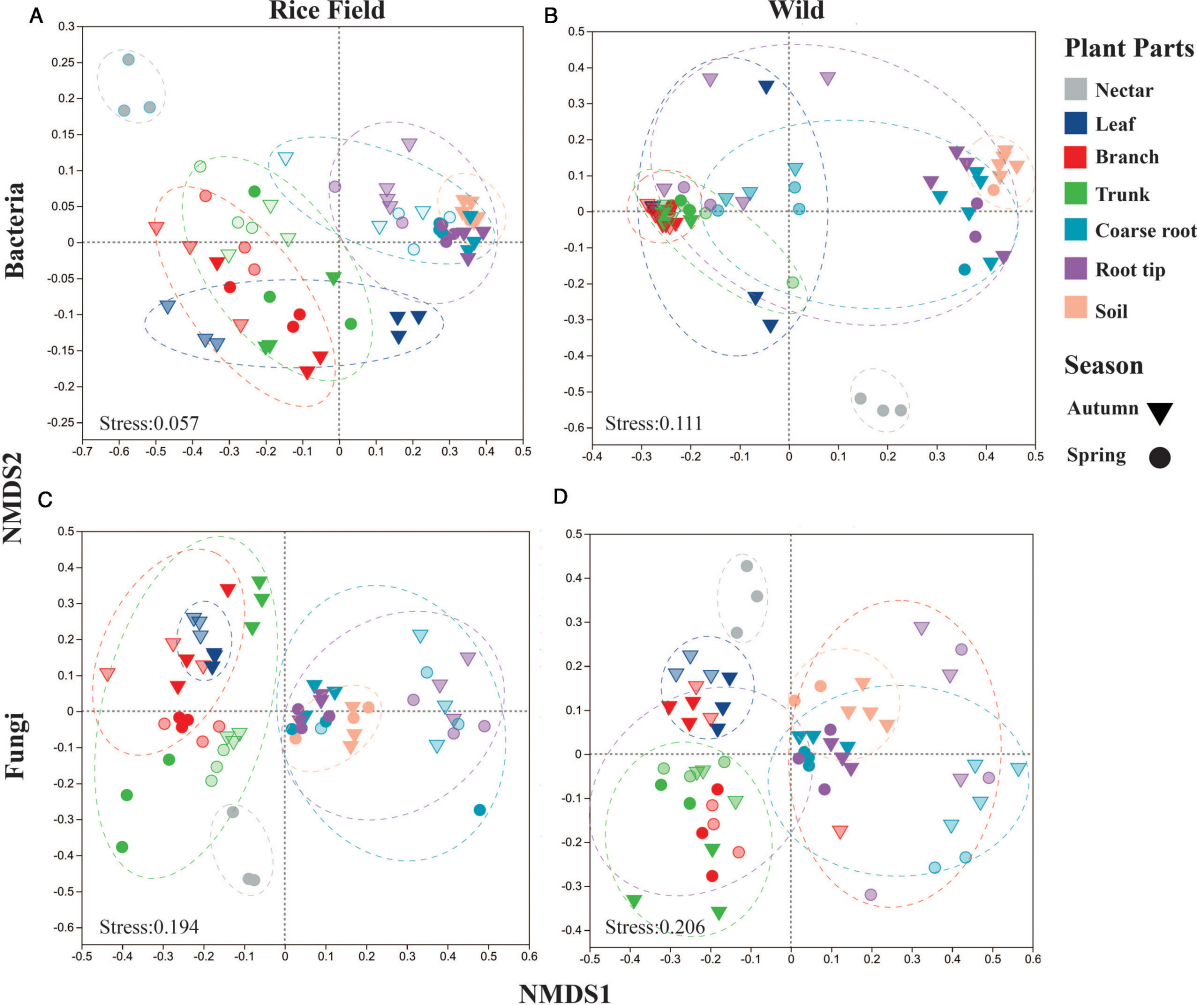
NMDS ordinations of both bacterial and fungal communinities across the plant parts (nectar, leaf, branch, trunk, coarse root, root tip, and soil) at different niches (endophytic and epiphytic), seasons (autumn and spring), and habitat (rice and wild). (**A** and **C**) The NMDS ordination of bacterial and fungal communities, respectively, in rice field grown *B. ceiba*. (**B** and **D**) The NMDS ordination of bacterial and fungal communities of *B. ceiba* grown in the wiild. The circle represents the spring season, while a triangle shape represents the autumn season. The filled color represents the epiphytic, and the light color represents the endophytic niche. Based on ANOSIM results, seasonal change influences bacterial and fungal community composition in different niches. Significance levels: stress value < 0.2.

Beta diversity analysis using NMDS and Bray-Curtis distances showed strong clustering by plant parts and seasons. Rice growing field of *B. ceiba* showed substantial variation in bacterial and fungal communities (*R* = 0.76 and *R* = 0.75) than wild habitat (*R*= 0.57 and 0.66), respectively in endophytic and epiphytic niches across different parts (nectar, leaf, branch, trunk, roots, and soil) and seasons (Fig. 4). Above-ground parts clustered separately from below-ground, and endophytic and epiphytic communities were clearly distinguished. The rice field showed more variation in above-ground parts across seasons than the wild habitat, which had more consistent clustering. There was apparent microbial clustering in different niches, as seen in the ordination representation (Fig. 4) with a parallel observation in the supplementary data analysis (see Fig. S5 at https://doi.org/10.5281/zenodo.17585670), along with the ANOSIM test indicating a significant statistical result of seasonal and habitat effect on plant parts (Table 1).

**TABLE 1:** The seasonal and habitat effects on the bacterial and fungal community structure across all plant parts were calculated using ANOISM^*a*^

Field	Plant part	Season	Niches	Bacteria		Fungi	
				*R*	*P*	*R*	*P*
Rice field	Nectar	Spring		0.592	0.098	0	0.296
	Leaf	Autumn	Endo vs phyllo	1	0.091	0.296	0.19
	Branch	Autumn vs spring	Endo vs phyllo	0.376	0.03	0.052	0.26
	Trunk	Autumn vs spring	Endo vs phyllo	0.574	0.002	0.274	0.054
	Coarse root	Autumn vs spring	Endo vs phyllo	0.577	0.002	0.163	0.13
	Root tip	Autumn vs spring	Endo vs phyllo	0.0675	0.003	0.132	0.14
	Soil	Autumn vs spring		−0.148	0.896	−0.037	0.59
	All			0.76	0.001	0.75	0.001
Wild	Nectar	Spring		0.059	0.098	0	0.296
	Leaf	Autumn	Endo vs phyllo	1	0.091	−0.037	0.507
	Branch	Autumn vs spring	Endo vs phyllo	0.345	0.043	0.45	0.006
	Trunk	Autumn vs spring	Endo vs phyllo	0.031	0.47	0.172	0.11
	Coarse root	Autumn vs spring	Endo vs phyllo	0.632	0.003	0.42	0.021
	Root tip	Autumn vs spring	Endo vs phyllo	0.666	0.003	0.083	0.24
	Soil	Autumn vs spring		0.148	0.291	0.29	0.19
	All			0.57	0.001	0.66	0.001

^*a*^ Analysis of similarity with Bray Curtis-distance matrix method (999 replacements). Significance levels: *P* < 0.05. *R*, ANOISM test statistic.

### Shared OTUs

**Fig 5:**
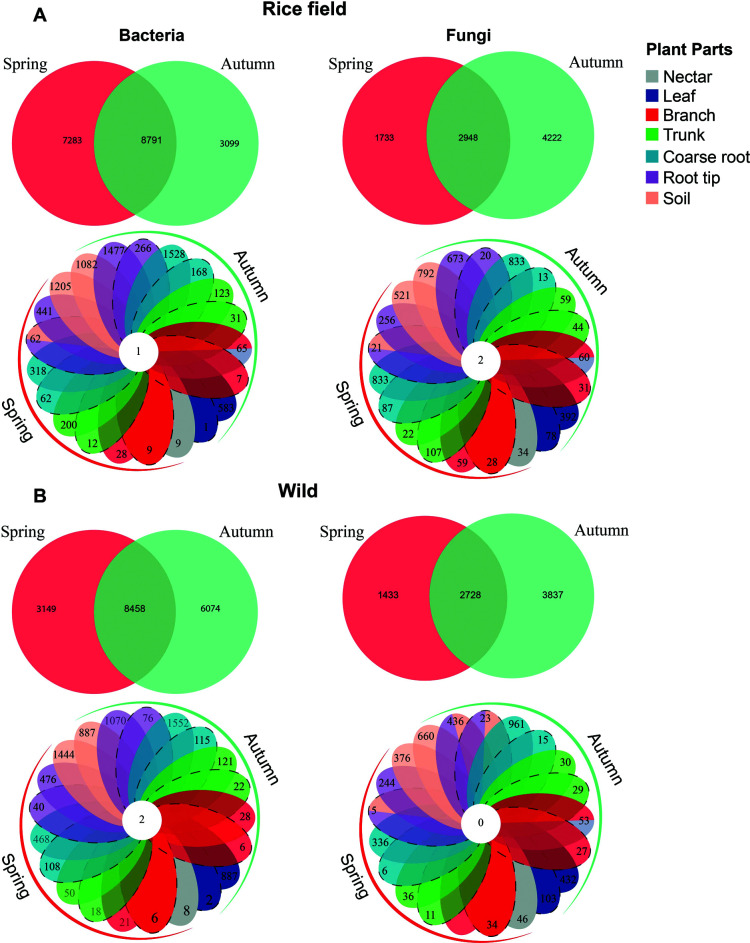
Venn diagram of the number of OTUs obtained in different parts at different niches during spring and autumn in different habitats. Values represent the numbers of OTUs; different colors represent the specific parts (dark colors represent epiphytes, dotted boundaries represent endophytes), and seasons. (**A**) The overall bacterial and fungal communities across different seasons and niches in the rice-growing field of *B. ceiba*. (**B**) The bacterial and fungal communities across different seasons and niches in the wild habitat of *B. ceiba*.

The amount of shared bacterial and fungal OTUs was found to be higher in the rice habitat (*n* = 8,791 and *n* = 2,948) than in the wild (*n* = 8,458 and *n* = 2,728) (Fig. 5). The number of shared bacterial and fungal OTUs between different parts in rice habitat (*n* = 1 and *n* = 2) was different from wild habitat (*n* = 2 and *n* = 0). Seasonal change showed a significant effect on several bacterial OTUs in both habitats. The spring season was found to have much an abundance of OTUs in the rice habitat (*n* = 7,283) than in autumn (*n* = 3,099) (Fig. 5A). However, in the wild habitat, the number of OTUs was found to be higher in autumn (*n* = 6,074) as compared to spring (*n* = 3,149) (Fig. 5B). Seasonal change showed significant results on the number of fungal OTUs in both habitats. The autumn season was found to have higher numbers of OTUs in rice and wild habitats (*n* = 4,222 and *n* = 3,837) than spring (*n* = 1,733 and *n* = 1,733) in wild habitats (Fig. 5). In conclusion, it was observed that seasonal change had effects on several OTUs in both habitats.

### Microbe source tracking

Microbial source tracking (MST) revealed that bacterial and fungal communities in both endophytic and epiphytic niches of *B. ceiba* were partially derived from soil and phyllosphere (aerial), with gradual microbial filtering across plant parts in both rice field and wild habitats. Nectar-associated microbes were mainly sourced from the root zone, suggesting internal translocation via xylem or phloem.

For fungi, 33% and 25% of OTUs in the root tip and coarse root rhizospheres, respectively, originated from soil and filtered into endophytes. Above-ground fungal communities also largely originated from unknown sources, with evidence of bidirectional filtering between epiphytic and endophytic niches—particularly in leaves, where 97% of OTUs were shared. In spring, trunk endosphere and unknown sources were the primary contributors to fungal OTUs in nectar (see Fig. S6 at https://doi.org/10.5281/zenodo.17585670).

### Habitat and seasonal influence on community network of *B. ceiba*

**Fig 6:**
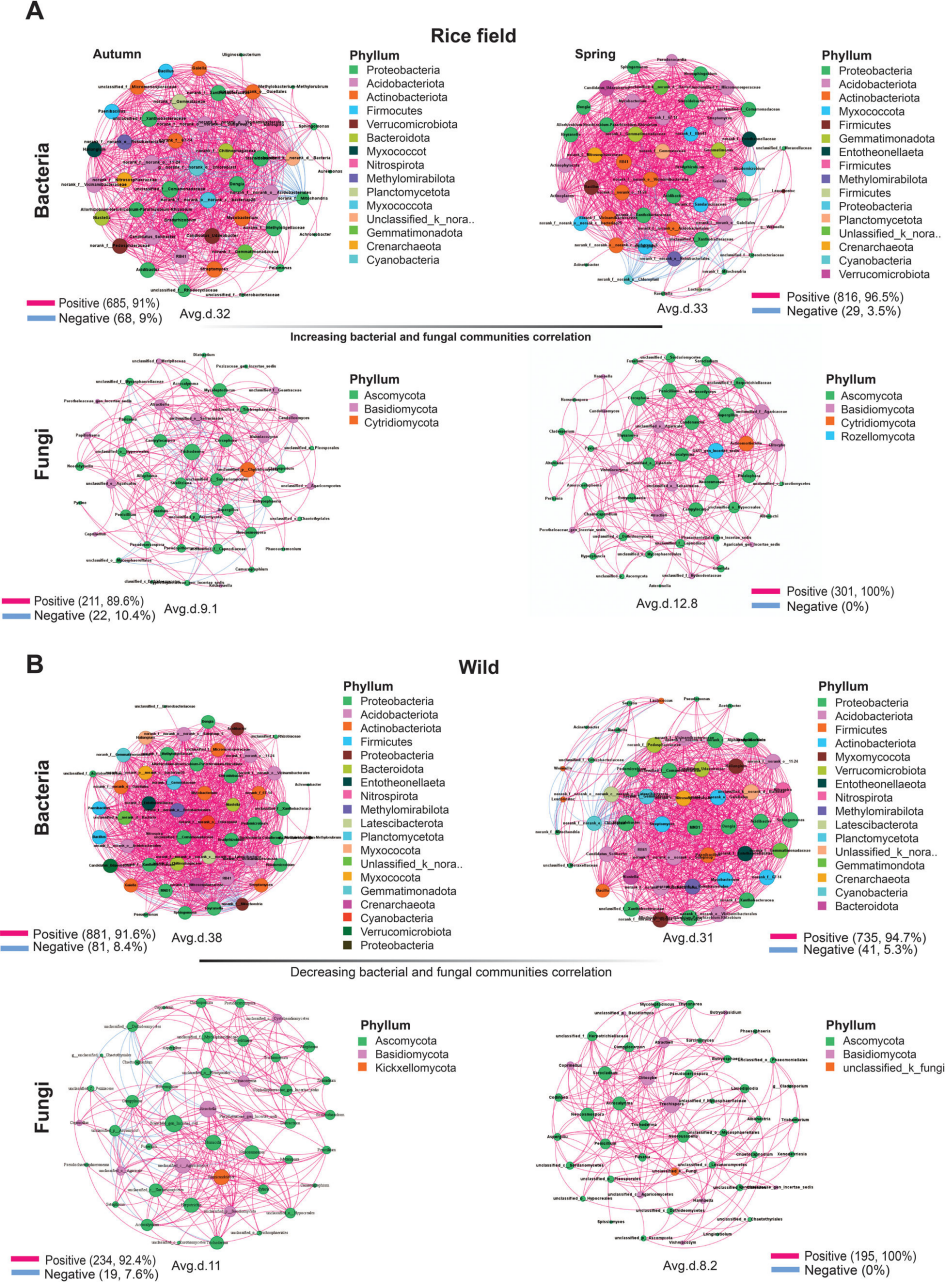
Correlation network study of microbial populations in various habitats and seasons. (**A**) Diagram depicting the bacterial and fungal community correlation network in the rice growing field of *B. ceiba*. (**B**) The bacterial and fungus communities' correlation network in the wild. Node color corresponds to the phylum to genus classification. Larger nodes indicate higher relative abundances, and edge color distinguishes between positive (red) and negative (blue) associations. These features highlight the non-random organization of microbial communities, with certain taxa acting as potential hubs.

A correlation network analysis was performed to investigate the intricacy of the relationships among the microbial communities of *B. ceiba* across different habitats and seasons, and the network’s topological characteristics were computed. Spring samples showed a high degree of complexity and modularity (Fig. 6A), with a large number of connections (average degree/node = 33), in contrast to the autumnal samples (average degree/node = 32) for rice growing fields of *B. ceiba*. Meanwhile, in the wild, autumn samples were found to have a higher degree of complexity and modular structure (Fig. 5B), with a node (average degree/node = 38) relative to the spring season (average degree/node = 31). A similar correlation network was found for fungal communities across both habitats in different seasons. The spring season had more fungal complexity than the autumn season in rice-growing fields of *B. ceiba* (Fig. 6A). Conversely, the wild habitat had inverse results compared to the rice habitat (Fig. 6B). In summary, the correlation network analysis showed that habitat and seasons significantly impacted the degree of microbial community interaction across all plant parts.

### Relative abundance of functional fungal guild

**Fig 7:**
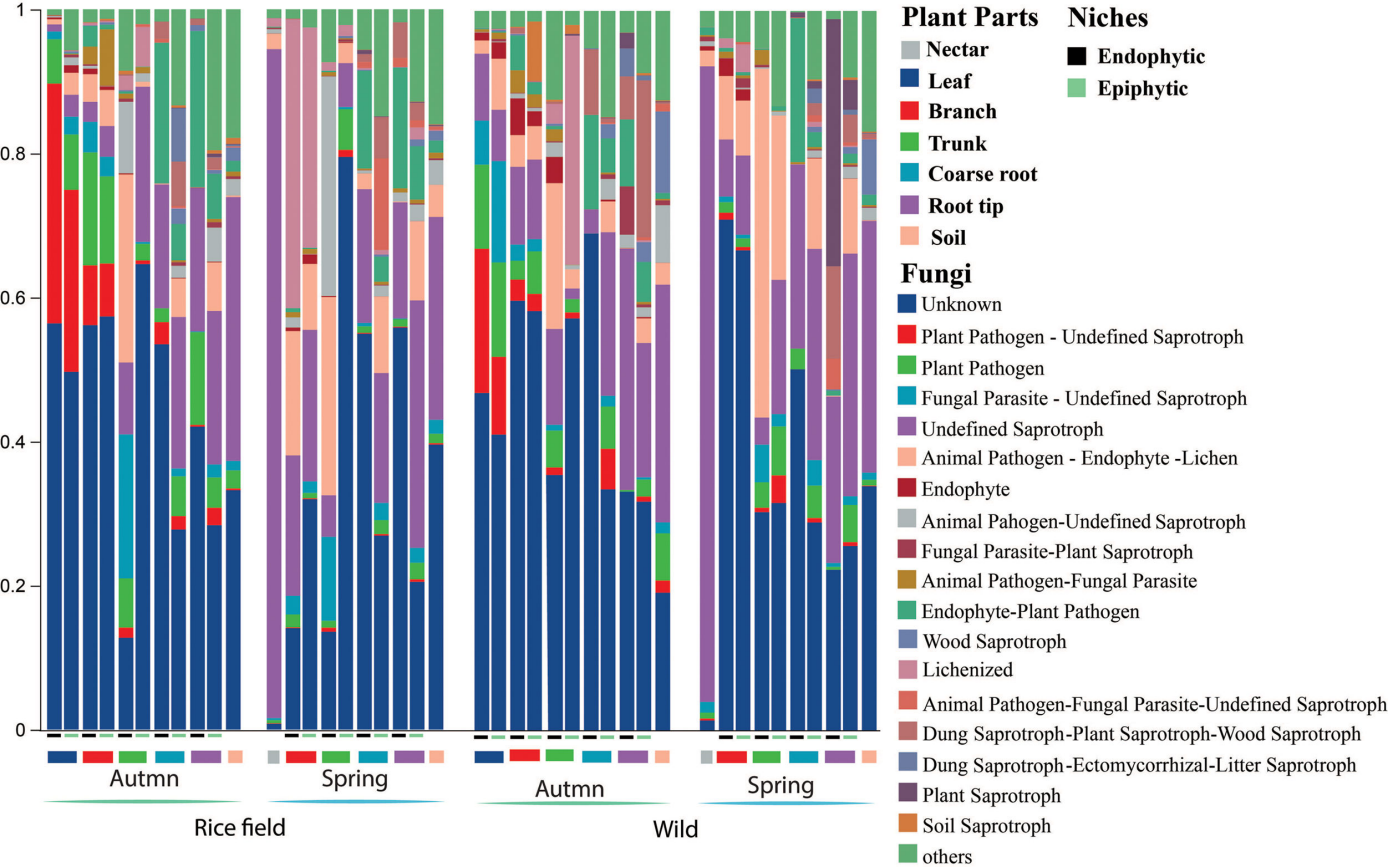
Relative abundance of fungal functional groups (guilds) inferred by FUNGuild at endophytic and epiphytic niches of plant parts across different habitats and seasons. Others merge < 0.01.

The most predominant funguild in wild and rice-grown fields of *B. ceiba* were unknown (0.43 and 0.46) and undefined saprotrops (0.144 and 0.146) in autumn, collectively accounting for at least 0.57 and 0.606, respectively, of the total funguilds (see Fig. S7 at https://doi.org/10.5281/zenodo.17585670). However, in spring, the relative abundance of the unknown guild decreased compared to autumn. In spring, the most predominant fun guild was unknown (0.33 and 0.34), undefined saprotrophs (0.26 and 0.27), and wood saprotrophs (0.08 and 0.11), collectively accounting for at least 0.67 and 0.72 (see Fig. S7 at https://doi.org/10.5281/zenodo.17585670). Moreover, the significant distribution of funguilds at endophytic and epiphytic niches of all plant parts was observed as shown in (Fig. 7).

### PICRSt2 function prediction

**Fig 8:**
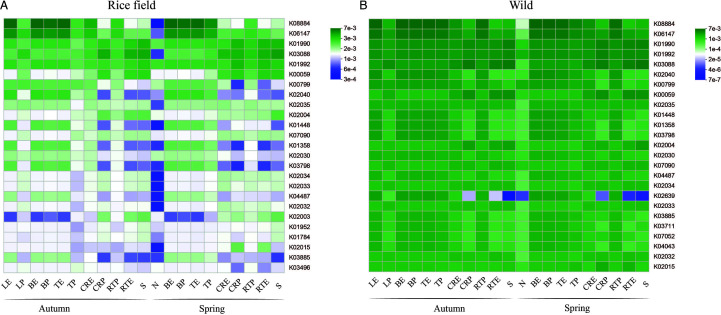
PICRUSt2 functional profile of *Bombax ceiba* bacterial microbiome. (**A and B**) Heat map exhibiting the relative abundance of KO in different habitats (rice and wild) and seasons (spring and autumn) across all plant parts. LE, leaf endosphere; LP, leaf phyllosphere; BE, branch endosphere; BP, branch phyllosphere; TE, trunk endosphere; TP, trunk phyllosphere; CRE, coarse root endosphere; CRP, coarse root phyllosphere; RTE, root tip endosphere; RTP, root tip phyllosphere; S, soil; N, nectar.

The functional potential of microbial communities in Bombax ceiba across rice and wild habitats and different seasons, predicted using PICRUSt2, showed distinct differences in KEGG Ortholog (KO) abundance. Wild habitats exhibited a significantly higher abundance of KO functional groups compared with rice fields, with variation observed across plant niches and seasons (Fig. 8). In *B. ceiba*, the functional genes K08884 (serine/threonine kinase) and K06147 (ABC transporter) were significantly abundant in the above-ground endosphere (leaf, branch, trunk) in both rice and wild habitats. In contrast, genes K01990, K03088, and K01992 were more abundant in below-ground parts (roots and soil). These patterns highlight the functional specialization of microbial communities across plant parts and habitats, suggesting a strong plant–microbiota–environment interaction that supports plant health and adaptability.

### PLS-PM model to study the influence of seasons and niches on microbial assembly and functional potential of *B. ceiba*

**Fig 9:**
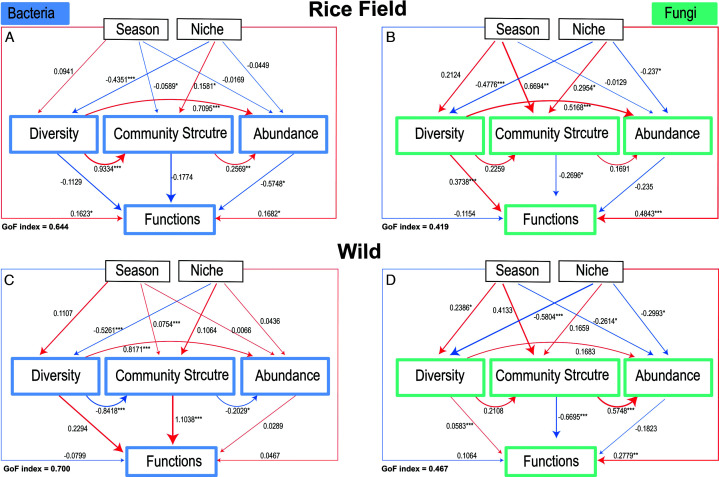
Structural equation model showing the impacts of season and niches on microbial diversity, structure, and functions across different habitats. (**A–D**) The SEM model for rice and wild habitat of *B. ceiba* for bacteria and fungi, respectively. Red lines represent the positive, and blue lines represent the negative relation. The width of the arrows indicates the strength of standardized path coefficients (*P < 0.05; **P < 0.01; ***P < 0.001).

A partial least squares path modeling (PLS-PM) analysis revealed how season and niche influence microbial diversity, community structure, abundance, and functional potential across rice-grown and wild habitats of *B. ceiba* (Fig. 9). The model fit (GoF) was stronger for bacterial communities (rice: 0.644; wild: 0.700) than for fungal communities (rice: 0.419; wild: 0.467), suggesting better structural representation of bacterial dynamics (Fig. 9).

## DISCUSSION

The microbiota of plants is integral to ecosystem functioning, influencing plant health, nutrient cycling, and resilience to environmental stress. Understanding the processes that shape these microbiomes, particularly across different niches and environmental contexts, is essential for unraveling their ecological roles. Seasonal dynamics and habitat characteristics are increasingly recognized as critical factors driving microbial assembly, yet their interactions remain poorly understood in many plant species. This study contributes to bridging this gap by exploring how microbial communities associated with *B. ceiba* respond to seasonal variations and habitat differences across diverse plant niches. The dynamic interplay between seasonal changes and habitat types plays a pivotal function in forming the microbial communities connected to *Bombax ceiba* across various plant niches.

Initially, we estimated alpha diversity, and it was discovered that the microbial community diversity was highly dependent on plant parts of flower, leaf, branch, trunk, and roots, which were clearly distinguished from one another by lowering OTU richness and diversity from below-ground parts towards above-ground parts (see Fig. S3 at https://doi.org/10.5281/zenodo.17585670). This rapid diversity loss from soil to roots, followed by shoot and flower, suggests that host-specific attributes are becoming more and more influential at the interfaces between roots and soil and roots and shoots (31). The root zone exudation and rhizodeposition encourage chemoattraction and colonization of rhizosphere soil and rhizoplane, which leads to the formation of the structurally diverse, intensely colored, and diverse rhizosphere microbial community (6, 32). A similar trend of reduced richness and diversity was observed from roots and rhizosphere to soil, leaves, branches, and trunks within the phyllosphere compartments of the tree. However, bacterial richness and diversity were notably higher in the phyllosphere compartments than in the endosphere. This overall decline in microbial richness and diversity from soil to roots and other phyllosphere plant compartments can be attributed to increasingly harsher environmental conditions, which result in reduced microbial diversity as we move toward the upper plant compartments (5, 33).

Like bacteria, the epiphytic fungal population had a higher species diversity than the endophytic fungal community. Endophytes may have lower richness than epiphytes due to their more stable environment and protection from environmental conditions provided by plant tissues (34). Moreover, our findings also showed that microbial richness and diversity were highest during the autumn season, aligning with previous research. The increased richness of microbes in autumn could be explained by more beneficial ecological conditions. This is because high temperatures and low moisture stability contribute to a more stressful environment within which microbial growth may occur (35). Moreover, inputs of organic matter in the form of leaf litter are sources of more diverse substrates that support the growth of a broader range of microbial taxa (s) (36). And these stable conditions favor the development of more diverse microbial communities. Due to resource availability and good climatic conditions, the life cycles of specific microbial species peak in the autumn, increasing the season’s overall variety and richness (37).

A microbial source tracking study revealed that soil was the predominant microbial source for the root endosphere, whereas aerial niches had primarily unknown sources, most likely indicating airborne deposition, rain splash, or insect vectors. This is consistent with the sequential filtering concept, in which microorganisms progress from bulk soil to rhizosphere to root endosphere, with greater selection at each stage. (38). MST also revealed vertical microbial translocation, with nectar microorganisms tracing back to root zones, indicating movement via the xylem and phloem. Although our study indicates that belowground microbes may have a role in contributing to nectar communities, this does not represent a confirmed route, but merely a hypothesis at this point. However, systemic microbial movement has been seen in several species, indicating symplastic or apoplastic transport of microorganisms or their propagules (39).

Furthermore, our finding indicates that the rice field showed a significant variation between plant compartments and seasons compared with the wild environment of *B. ceiba* by NMDS analysis (Fig. 4). It may be due to environmental heterogeneity; rice growing fields have a more diverse environment due to the different practices in fields. This practice can create diverse microhabitats that support a diverse array of species. These results align with earlier research that shows how the environment of plants shapes the assembly of plant-associated microbiomes in various niches (40).

The allocation of every OTU that has been found throughout the various plant parts also highlighted several other aspects of the study: (i) large number of OTUs in the spring, (ii) the obligate endophytes which can be found only in the endosphere of plant tissues and which are as important to plant survival as the plants themselves (40), (iii) existence of facultative endophytes which are found on the phyllosphere and endosphere of the plant parts, and (iv) however, most of the endophytic bacteria that infect the host plant are derived from the rhizosphere of the plant (41), some could come from out of the environment (for example, aerosols colonizing the phyllosphere and then the leaf endosphere) (42), as demonstrated by the proportion of OTUs uniquely identified in the leaf endosphere and phyllosphere samples (e.g., 0.01% and 3.04% and 0.01% and 5.02% in rice and wild environment of *B. ceiba*. However, fungal OTU distribution in leaf endosphere and phyllosphere was found to be high as compared with bacteria (e.g., 0.88% and 4.4% and 1.29% and 5.41%) in rice and wild environments, respectively (Fig. 5). This could be due to the high resistance desiccation of fungal communities compared with bacteria, as fungi are more resilient to ultraviolet radiations on leaf surfaces, especially in exposed conditions. Moreover, many fungi have developed distinctive plant interactions, such as pathogenic and symbiotic. This characteristic usually results in more abundant than bacterial communities (43).

The dominant bacterial phyla were Proteobacteria, Cyanobacteria, Firmicutes, and Acidobacteria. Proteobacteria were most abundant in phyllosphere niches, while Cyanobacteria dominated the endosphere, consistent across both rice and wild habitats regardless of season. Firmicutes and Acidobacteria were mainly present in below-ground parts (roots and soil). Notably, Firmicutes showed increased relative abundance during the spring, particularly in the rhizosphere and nectar (Fig. 2). Our results showed that the high relative abundance of these phyla in the rhizosphere follows those of poplar (44) and moso bamboo (45) and revealed that the formation of *B. ceiba* rhizosphere bacterial communities also obeys the general regularities of microbial wire formation. Among these, Proteobacteria and Acidobacteria are two key types of rhizobacteria. It has been shown that the quantity of Proteobacteria and Acidobacteria in rhizobacteria is a good way to find out the amount of nutrients in the soil; Proteobacteria are linked to soil that is rich in nutrients, while Acidobacteria are linked to soil that is low in nutrients (46). Like the primary makeup of rhizosphere soils, such as those of poplar (44) and bamboo (47), the relative abundance of Proteobacteria increased in the root endosphere, while the relative abundance of Acidobacteria decreased from the rhizosphere soil to the root microbiota. The enrichment of these species in rhizosphere soil and root endosphere was consistent across rice and wild habitats, demonstrating that these bacterial communities are abundant around the *B. ceiba’s* roots.

We also found that *Bacillus* belongs to the Firmicutes phylum, a bacterial genus known to include plant growth-promoting rhizobacteria (PGPR) that change in response to N content (48). The quantity of *Bacillus* in the rhizosphere soil and plant tissues during the spring (flowering) is higher than autumn (nonflowering), indicating that the spring season may get a more accessible nitrogen supply. Moreover, more Bacillus was found in the samples taken from the rice grown in *B. ceiba* than in the wild. Furthermore, more studies on these rhizosphere-specific bacteria will assist in better understanding the activity of bacteria connected to *B. ceiba*.

Environmental influences on the fungal composition of phyllosphere and endosphere niches in all plant sections have not been well studied. We found that the epiphytic fungal community in below-ground parts (roots) was dominated by Dothideomycetes and Sordariomycetes, in conformity with other findings on tropical and temperate plant species (49). The Dothideomyctes is acknowledged as having the most extensive and varied ecological and functional features. This group consists of several species regarded as the pathogens of both human beings and plants, as well as endophytes and epiphytes. Furthermore, it is often known that Dothideomyctes is one of the significant taxa associated with leaves (50), which is consistent with our study because the highest abundance of Dothideomyctes was found in the leaves and continues to decrease from above-ground parts towards lower parts of the tree. We also found that the above-ground part (branch and trunk) phyllosphere was highly abundant with the unclassified_k_fungi. The branch and trunk endophytic niches are Dothideomyctes. A similar pattern of fungal communities’ distribution was observed in both environments (rice field and wild) of *Bombax ceiba*; however, as expected, seasonality had a more significant influence on epiphytic than on endophytic fungal community structure.

Furthermore, using the normalized stochasticity ratio (NST) to examine microbial community assembly reveals divergent patterns across bacterial and fungal species across plant niches, seasons, and habitat types. Bacterial communities in endophytic habitats had lower NST indices, indicating the dominance of deterministic processes like host filtration and nutrient selectivity. Epiphytic bacterial communities, on the other hand, demonstrated greater stochasticity, particularly during the fall season, owing to increased environmental variability and dispersal constraints. These findings are consistent with earlier research indicating increased environmental selection within host tissues, while more changeable circumstances favor stochastic assembly (45). Furthermore, bacterial communities in wild environments were more stochastic than those in rice fields, demonstrating the impact of habitat complexity on neutral processes.

In contrast, fungal communities consistently had higher NST indices throughout all plant sections and seasons, demonstrating the importance of stochastic processes like random colonization and spore dissemination. This pattern was especially noticeable in wild environments and epiphytic niches, where neutral assembly mechanisms are probably encouraged by the unpredictable environment and absence of host-imposed filters (51). The observed seasonal variation highlights the importance of temporal dynamics in the structure of microbial communities, with the maximum stochasticity in both bacterial and fungal communities occurring in the autumn. These findings are consistent with a niche-neutral continuum model (52), where the microbial group, plant compartment, and environmental setting all influence the relative dominance of deterministic and stochastic forces.

To better understand how microbial community members interact, network analysis using correlation tests has become increasingly popular in recent years. In our findings, the co-occurrence network shaped by the bacterial and fungal communities continued to exhibit seasonal variations across different habitats. For example, in the spring (flowering) season in rice fields, there was an increase in the interactions observed in the bacterial and fungal communities compared with autumn. On the other hand, similar trends were revealed as anti-correlated with the wild environment of *B. ceiba*; the bacterial and fungal communities had lower interaction in the spring than in autumn. To prepare rice fields for planting, fertilizers and other nutrients for the soil are added in the spring. A more conducive environment for microbial development and activity is produced by this increase in soil nutrients, especially nitrogen (N), phosphorus (P), and potassium (K). By interacting with the soil microbiome, *B. ceiba* contributes to increased microbial diversity and activity throughout this time, further enhancing the soil’s nutritional content (15). Moreover, dynamic plant-soil-microbe interactions may be produced by the phenological phases of rice and the springtime flowering of *B. ceiba*. Both *B. ceiba* and rice produce more root exudates, which serve as organic carbon sources for a varied microbial population. As microorganisms interact with the root exudates with one another, the resulting microbial networks become increasingly intricate and linked (53). These elements work together to make the microbial networks surrounding *B. ceiba* trees in rice fields more complicated and correlated during the spring (Fig. 6).

Moreover, our results suggest that plant microbiome composition and potential PICRUSt2 functions change across endophytic and epiphytic niches according to environmental dynamics. As PICRUSt2 is a predictive tool, the functional roles reported here are inferred based on marker gene profiles rather than directly measured. In the current study, we examine that the rice-growing field of *B. ceiba* had a low abundance of functional groups KOs as compared to the wild environment (Fig. 8), which relates to the previous studies that the human-mediated agricultural methods have a big effect on the cultivated crop plant’s rhizobiome, which frequently causes the rhizosphere’s native microbial population to be disrupted and altered (54, 55). Moreover, a more varied microbial population is usually seen in wild settings because of the ecosystem’s complexity and diversity. Because different microorganisms provide diverse roles, this variety may result in more functional genes. In contrast to agricultural fields, which may be impacted by nutrient depletion and artificial fertilization, wild ecosystems frequently have a more natural and balanced nutritional profile. This natural nutrition availability supports various microbial activities and functions (56).

Furthermore, SEM analysis demonstrates vital information about bacterial and fungal microbial assembly and functional potential in *B. ceiba* from rice fields and wild areas through its results (Fig. 9). Overall, wild microbial communities displayed more stability and functional integration when compared with rice field environments, since agricultural practices and seasonal variations affect microbial variability. Natural ecosystems support stable microbial interactions that are essential for plant-microbe symbiosis because they show positive correlations of community structure with function in wild habitats (57). Conversely, the relationship between microbial abundance and function appears distinct in rice fields since anthropogenic disturbances can lead to diminished functional influence (58).

### Conclusion

The *B. ceiba* plant serves as an ideal model to explore microbial community dynamics across endophytic and epiphytic niches in different habitats. Our study reveals that epiphytic microbial communities exhibit greater structural variability than endophytic ones across plant parts (nectar, leaf, branch, trunk, roots, and soil), with environmental changes driving epiphytic shifts, while endophytic communities in leaves, branches, trunks, and roots remain relatively stable. Vertical stratification and seasonal transitions (flowering vs non-flowering) significantly shape microbiomes, with soil interactions playing a key role. Bacterial communities showed higher variability than fungi, suggesting niche-specific assembly processes. Wild habitats foster stable plant-microbe alliances, whereas rice fields disrupt microbial stability and function. Habitat preservation is critical for maintaining microbial diversity and ecosystem efficiency. By demonstrating how seasonal and structural gradients alter microbial composition, this study highlights how it is possible to manipulate plant-associated microbiomes to increase soil fertility without using chemicals, plant resilience, and maintain sustainable habitat and agroforestry systems. Future research should integrate plant physiology, precise taxonomic profiling, and soil properties to elucidate habitat-microbe linkages.

## Data Availability

The raw sequencing reads are available in the SRA database in NCBI (https://www.ncbi.nlm.nih.gov/) under the accession number SRP566300.
